# Co-Digestion of Olive Pomace and Goat Manure for *Hermetia illucens* Growth and Potential Coupling with Biogas Production

**DOI:** 10.3390/insects17050508

**Published:** 2026-05-16

**Authors:** Antonio Dolce, Giovanni Lomonaco, Francesco Iannielli, Nazaret Rubiejo Perez, Carmen Scieuzo, Jesus D. Fernandez Bayo, Patrizia Falabella

**Affiliations:** 1 Department of Basic and Applied Sciences, University of Basilicata, Via dell’Ateneo Lucano 10, 85100 Potenza, Italy; antonio.dolce@unibas.it (A.D.); giovanni.lomonaco003@unibas.it (G.L.); francesco.iannielli003@unibas.it (F.I.); carmen.scieuzo@unibas.it (C.S.); 2 Department of Soil Science and Agricultural Chemistry, Faculty of Pharmacy, University of Granada, 18071 Granada, Spain; nazaretrubiejo@correo.ugr.es; 3 Spinoff XFlies s.r.l., University of Basilicata, Via dell’Ateneo Lucano 10, 85100 Potenza, Italy

**Keywords:** anaerobic digestion, bioconversion, *Hermetia illucens*, biogas, frass

## Abstract

Olive pomace is an abundant agro-industrial residue whose recalcitrant composition limits its efficient valorisation, highlighting the need for sustainable recovery strategies. This study investigated the use of *Hermetia illucens* larvae for the bioconversion of olive pomace and its integration with anaerobic digestion to evaluate the simultaneous recovery of biomass and energy. Larvae were initially reared on olive pomace, goat manure, and their mixture to assess growth performance, survival, and substrate suitability. Subsequently, olive pomace was subjected to thermal pre-treatment and anaerobic digestion with goat manure, and the resulting digestates were reused as substrates for larval rearing. Their phytotoxicity was also evaluated through germination tests on alfalfa. Larval performance was higher on non-digested substrates, confirming the suitability of raw mixtures for insect rearing. Thermal pre-treatment did not result in a significant increase in methane production. In contrast, digestates, particularly those obtained under more severe thermal treatments, resulted in reduced larval growth and survival under the tested conditions and showed variable phytotoxic effects. Overall, although anaerobic digestion enabled energy recovery, its integration with insect-based bioconversion resulted in reduced larval performance under the tested conditions. These findings highlight the importance of optimising substrate treatment to ensure effective integration of insect rearing within circular bioeconomy systems.

## Simple Summary

Nowadays, the sustainable management of agro-industrial residues is one of the major environmental challenges. Olive pomace and animal manure are abundant materials that often pose disposal problems, but they can become valuable resources if properly managed. In this study, we evaluated an integrated approach to convert these wastes into useful products. First, we applied anaerobic digestion, a biological process that occurs in the absence of oxygen, to produce biogas, a renewable energy source. Then, we tested the remaining material as a growth substrate for black soldier fly larvae, insects capable of converting organic waste into high-value biomass. The results showed that biogas production was feasible, but thermal pre-treatment of olive pomace did not significantly improve yields. In addition, the digested residue, especially after heat treatment, was not suitable for optimal larval growth. This study highlights both the potential and the limitations of integrating energy production and insect-based bioconversion of organic waste, contributing to the development of more efficient circular economy strategies and to the reduction in environmental impacts.

## 1. Introduction

The growing need for sustainable management of organic waste and production of renewable energy has stimulated interest in approaches based on the circular bio-economy [1]. Agricultural and agro-industrial residues, such as olive pomace and animal manure, are rich in organic matter and can be valorised through biological processes, reducing environmental impact and generating high-value products [2].

### 1.1. Biological Processing of Organic Residues

Anaerobic digestion (AD) is a widely used technology that allows the recovery of biogas (a mixture mainly composed of methane and carbon dioxide) from organic substrates under oxygen-free conditions [3]. In addition to energy recovery, anaerobic digestion produces a digestate that can potentially be used as fertiliser, depending on its stability and nutritional content [4]. In parallel, bioconversion by insects, in particular the larvae of the black soldier fly (BSF), *Hermetia illucens*, is emerging as a promising strategy for the valorisation of organic waste. BSF larvae can transform waste into high-value biomass (protein and lipids) and frass, a mix of insect excretion and the residual substrate [5]. BSF larval frass is attracting increasing attention for its potential as a biofertiliser due to its nutrient content and possible presence of beneficial microorganisms [6,7,8]. Although anaerobic digestate can contain residual organic matter and nutrients, its biochemical stability and immediate nutrient availability can be limited without further processing, which may impact its suitability as a rearing substrate for *H. illucens*. Nevertheless, several studies have shown that even partially stabilised digestates can still provide sufficient macro- and micronutrients to sustain the growth of BSF larvae, especially when supplemented or pre-treated to improve nutritional quality [9,10].

### 1.2. Olive Pomace as Valorisable Substrate

However, there are still a few studies systematically analysing the combined application of these technologies, in particular using agro-industrial by-products, such as olive pomace [11]. Olive oil extraction results in the generation of large quantities of solid and liquid by-products, since only about 15–25% of the olive fruit mass is converted into oil while the remaining 75–85% becomes residues such as olive pomace and mill wastewater. Mediterranean countries account for the majority of global olive oil production. Spain typically contributes around 24–42% and Italy around 11–17% of total output, leading to enormous amounts of by-products produced annually during the short harvest season. These residues pose significant environmental management challenges due to their high organic load, phytotoxicity, and the costs and complexity associated with their proper treatment and disposal [12]. Olive pomace is the main solid by-product of olive oil production, consisting of skins, pulp, fragments of pit, and residual oil and water. It is rich in complex carbohydrates, residual lipids, and phenolic compounds, which provide antioxidant properties but may inhibit certain microbial processes. Due to its high organic content, olive pomace represents a valuable substrate for anaerobic digestion and bioconversion using *H. illucens*, as two promising and complementary pathways [5]. However, the recalcitrant nature of olive pomace, linked to its lignocellulosic structure and high content of phenolic compounds, often limits process efficiency, inhibiting microbial activity during anaerobic digestion and reducing substrate suitability for insect bioconversion. Olive pomace is primarily disposed of through land spreading, composting, or, in some cases, uncontrolled accumulation near processing facilities, which may lead to environmental concerns due to its high organic load and phytotoxic compounds. These constraints have prompted the investigation of various pre-treatment strategies aimed at improving substrate degradability and enhancing its valorisation in subsequent biological processes [13]. Among biological treatments, thermal pre-treatment is a simple and potentially economical solution, particularly in agricultural or rural contexts, where waste heat may be readily available and applied [14]. Thermal treatments can promote the solubilisation of organic matter and partial disruption of lignocellulosic structures, potentially improving anaerobic digestion [13]. Nevertheless, the actual effectiveness of thermal pre-treatment on olive pomace remains controversial. Excessive temperatures may lead to the formation of inhibitory compounds and variable effects on biogas production and downstream bioconversion efficiency, including BSF larvae growth performance [15]. Moreover, animal manure, that is commonly managed through direct land application or storage prior to use as fertiliser, with limited stabilisation, potentially resulting in nutrient losses and emissions if not properly handled, is commonly used as inoculum in anaerobic digestion processes due to its rich and well-adapted anaerobic microbial community, which enhances process start-up and methane production, particularly in the treatment of agricultural residues [16].

### 1.3. A Two-Stage Bio-Refinery Approach

This study aims to investigate a two-stage bioconversion pathway designed to maximise the valorisation of two widely available agro-zootechnical residues: olive pomace and goat manure. In the first stage, the bioconversion process was evaluated using these residues as feeding substrates. Based on the results obtained from preliminary trials, a mixture of olive pomace and goat manure in a 2:1 ratio was also tested, while a standard diet was adopted as a positive control.

Given the large availability of these by-products and the need to identify sustainable management strategies, an alternative valorisation pathway for the starting materials was also explored. In particular, based on the outcomes of the bioconversion trials, biogas production through anaerobic digestion was considered. Goat manure was applied as the starting inoculum, and the effect of thermal pretreatment at different temperatures on the anaerobic digestion performance of olive pomace was evaluated. Furthermore, the potential use of the resulting digestate as a potential feeding substrate for *H. illucens* larvae was investigated, with the aim of integrating renewable energy production with the generation of larval biomass.

The last assessed aspect in this study was the evaluation of the potential phytotoxic effects of the frass, obtained from the bioconversion assay, and of the obtained digestates. This assessment was conducted using alfalfa (*Medicago sativa*) as a model plant species, in order to evaluate the potential future use of these organic materials as fertilisers.

The novelty of this research lies in the proposal of an integrated cascading system that combines anaerobic digestion (AD) with insect bioconversion, specifically investigating the use of the resulting digestate as a direct feeding substrate for *Hermetia illucens* larvae. While many studies treat these processes separately, this work explores the synergy between renewable energy production and biomass generation to close the nutrient loop.

## 2. Materials and Methods

### 2.1. Raw Materials Characterisation

Raw materials used in the experiments were olive pomace, provided by the olive oil producer El Aceite de Casa Grande (Villanueva de la Reina, Spain), and goat manure, provided by a local farmer near the city of Granada (Spain).

The provided substrates were subjected to chemical-physical analyses to determine their main characteristics. Total Solids (TS) was measured by gravimetry, drying at 105 °C for 24 h and measuring the weight difference.

$$TS\;\left(\%\right)=\frac{Dry\;residue\;at\;105\;{}^{\circ}C\;(g)}{Fresh\;weight\;sample\;(g)}\times 100$$

To measure Volatile Solids (VS), the individual raw materials were dried at 105 °C for two and a half hours, cooled in a dryer for 30 min, calcined in a muffle furnace for 4 h at 550 °C, cooled again in a dryer for 30 min and finally weighed. After these steps, the percentage of volatile solids was calculated using the following formula.

$$VS\;\left(\%\right)=\frac{Dry\;weight\;at\;105\;{}^{\circ}C\;\left(g\right)-Dry\;weight\;a\;550\;{}^{\circ}C\;\left(g\right)}{Dry\;weight\;at\;105\;{}^{\circ}C\;\left(g\right)}\times 100$$

The pH and electrical conductivity (EC) were measured in a 1:5 (*w*/*v*) aqueous suspension stirred for 30 min, using a pH-metre and EC-metre (GLP31, Crison Instruments, Alella, Spain), respectively.

Laboratory protocols were used to measure nitrogen, phosphorus and potassium, using a photometer for nitrogen, a spectrophotometer for phosphorus and other steps to determine potassium [17].

### 2.2. Bioconversion Assay on Raw Materials

The bioconversion process was evaluated using the selected feedstock: manure alone, olive pomace alone, standard diet (chicken feed) as a control and a mixture of olive pomace and goat manure, in a 2:1 ratio (this ratio was chosen based on the results of preliminary tests). Four-day-old larvae provided by Biotecnologia Andaluza SL (Sevilla, Spain) were used for this test, and the parameters evaluated were:

(a) The survival rate (*SR*) was determined as the percentage ratio between the number of larvae alive at the end of the experiment and the initial number of larvae, where (*N_f_*) is the number of larvae alive at the end of the experiment and (*N_i_*) is the initial number of inoculated larvae, according to the following equation [18]:

$$SR\;\left(\%\right)=\frac{N_{f}}{N_{i}}\times 100$$

(b) The feed conversion rate (FCR) was calculated as the ratio between the larval biomass produced and the amount of substrate consumed, where (*B_f_*) and (*B_i_*) represent the final and initial larval biomass (dry weight), respectively, and (*S_c_*) is the amount of dry substrate consumed during the rearing period, expressed as a percentage [18]:

$$FCR=\frac{B_{f}-B_{i}}{S_{c}}$$

(c) The larval biomass percentage (BM) was calculated as the ratio between the final dry larval weight (*B_f_*) and the average final dry larval weight of the control (*C_f_*) [18]:

$$BM\;\left(\%\right)=\frac{B_{f}}{C_{f}}\times 100$$

(d) Substrate consumption (SC) was determined as the difference between the initial and final substrate mass as dry weight, where (*S_f_*) is the amount of residual substrate at the end of the experiment, *S_i_* is the amount of initial substrate and (*D_f_*) is the final dry weight of larvae [18]:

$$SC\;(\%)=\frac{S_{i}-S_{f}}{D_{f}}\times 100$$

The larvae were reared for approximately 9 days under controlled temperature (27–28 °C) and humidity (70%) conditions. All larvae were introduced with a similar initial fresh weight of approximately 0.02 g, with no statistically significant differences between the groups. At the end of the bioconversion, the excrement produced by each tray was subsequently collected, heat-treated at 70 °C for 1 h (as required by European Regulation 1925/2021) and used for the phytotoxicity test.

### 2.3. Anaerobic Digestion Assay

An additional objective was to evaluate biogas production from olive pomace subjected to different thermal pre-treatments under mesophilic conditions. The substrate was treated in an autoclave at 90, 120, and 150 °C for 1 h and 30 min to improve the solubilisation of organic matter and increase its degradability. Anaerobic digestion was performed using a mixture of pretreated olive pomace and goat manure (ratio 2:1, on a dry weight basis) at 70% humidity. Goat manure was used as inoculum for the mixture and alone as a negative control. Samples for AD tests were named as follows:

- A-E, non-treated olive pomace and goat manure;
- A90-E, olive pomace treated at 90 °C and goat manure;
- A120-E, olive pomace treated at 120 °C and goat manure;
- A150-E, olive pomace treated at 150 °C and goat manure.

Anaerobic digestion was performed using laboratory-scale reactors (1 L volume) under mesophilic conditions (constant temperature of 37 °C) for a period of 17 days [18]. Each reactor was connected to the OxiTop^®^ Control Multi 3630ID system (Xylem Analytics, Weilheim, Germany), which allowed the gas pressure to be measured in hPa.

#### Bioconversion Assay on Digstates

In addition, we evaluated the growth performance of *H. illucens* larvae reared using digestate as a feeding substrate. Prior to the bioconversion process, the pH of the different digestates was determined to evaluate their suitability for larval development. Larvae were provided by Biotecnologia Andaluza 2023 SL (Sevilla, Spain). At the end of the AD described above, four-day-old larvae were placed directly in each reactor, without any further processing on the obtained digestate, and reared for 9 days under controlled temperature and humidity conditions. No additional feed was provided during the test. To evaluate the biological performance of *H. illucens* larvae, the survival rate (SR), feed conversion rate (FCR), larval biomass percentage (BM), and substrate consumption (SC) were calculated. In addition, in this bioconversion trial, we measured the final larval weight to evaluate how the same substrate that had been further exploited for biogas production could affect larval growth.

### 2.4. CHNS Analysis

Total elemental CHNS (Carbon, Hydrogen, Nitrogen, and Sulfur) analysis was measured using an Elemental Analyzer Flash 2000 Model (Thermo Scientific, Milan, Italy) at the Scientific Instrumentation Centre of the University of Granada. These analyses were performed on frass (obtained from the two bioconversion assays) and on different produced digestates.

### 2.5. Phytotoxicity Tests

To evaluate the implications of possible agricultural applications, extracts of frass and digestates were produced to carry out phytotoxicity tests.

As starting material, the following samples were selected:

- frass obtained from bioconversion on standard diet;
- frass obtained from bioconversion on the mixture of olive pomace and goat manure (2:1);
- digestates obtained from the AD of the mixture of olive pomace and goat manure (2:1), in particular A-E and A120-E.

The two types of frass were selected based on data obtained from the bioconversion assay (e.g., better larval growth performances), while the digestates were selected for their ability to yield the highest biogas production. The extracts were prepared with a sample-water ratio of 1:10, stirred for 30 min at room temperature, centrifuged for 10 min at 10,000× *g* and finally filtered with filter paper (FILTER-LAB, ref. 1254, pore 2–4 µm). A layer of filter paper was placed in a 10 cm Petri dish and moistened with 5 mL of the extract. Ten alfalfa seeds (*Medicago sativa*) were placed in each Petri dish, and distilled water was used as a control [19].

The germination index (GI) was calculated based on the number of seeds that germinated and the total number of seeds placed in germination [20].

$$GI=\frac{n.\;seeds\;germinated\;in\;sample}{n.\;seeds\;total\;in\;sample}\times 100$$

The seed germination index (SGI) was calculated on the basis of the number of germinated seeds and the length of the rootlets, compared to a control on distilled water [20].

$$SGI=\left[\left(\frac{n.\;seeds\;germinated\;in\;sample}{n.\;seeds\;germinated\;in\;control}\right)\times \left(\frac{radicle\;length\;in\;sample}{radicle\;length\;in\;control}\right)\right]$$

### 2.6. Statistical Analysis

All experiments were performed in triplicate (three independent biological replicates), and results were expressed as mean ± standard deviation (SD). Statistical analyses were performed using GraphPad Prism 6.0 software (GraphPad Software, Inc., La Jolla, CA, USA). Data were analysed by one-way analysis of variance (one-way ANOVA) to assess differences among treatments. When significant differences were observed, Tukey’s multiple comparisons test was applied. Differences were considered statistically significant at *p* < 0.05.

## 3. Results

### 3.1. Characterisation of Raw Materials

Analyses were conducted on olive pomace, goat manure, and their 2:1 mixture. The results of the physio-chemical characterisation, including pH, electrical conductivity, organic carbon, organic matter, volatile solids, nitrogen, phosphorus, and potassium (on a dry matter basis), are reported in Table 1. Olive pomace showed an acidic pH (6.10 ± 0.01), whereas goat manure was alkaline (8.87 ± 0.03). The mixture exhibited a near-neutral pH (7.48 ± 0.02), suggesting a buffering effect between the two substrates. Electrical conductivity was highest in goat manure (13.19 ± 0.30 mS/cm), followed by the mixture (9.18 ± 0.07 mS/cm) and olive pomace (5.18 ± 0.05 mS/cm), indicating a higher salt content in the manure. Organic carbon and organic matter were higher in olive pomace (5.71 ± 0.93% and 9.85 ± 1.61%, respectively) compared to goat manure (3.62 ± 0.67% and 6.23 ± 1.16%), while the mixture showed intermediate values, reflecting the contribution of both substrates. A similar trend was observed for volatile solids. In contrast, nitrogen and phosphorus concentrations were higher in goat manure (2.30 ± 0.15% and 2.15 ± 0.22%, respectively) than in olive pomace (1.80 ± 0.25% and 0.36 ± 0.12%), whereas the mixture again showed intermediate values, indicating improved nutrient balance. Potassium content was higher in olive pomace (2.14 ± 0.09%) than in goat manure (1.71 ± 0.15%), with the mixture presenting values between the two substrates.

### 3.2. Bioconversion Assay on Raw Materials

Larval performance varied markedly depending on the substrate used for rearing (Table 2). The survival rate (SR) remained high across all treatments, ranging from 94.4% to 98.4%. The highest survival was observed in the standard diet (98.4%), followed by the olive pomace–goat manure mixture (98.3%) and goat manure alone (97.6%), while the lowest value was recorded for larvae fed exclusively with olive pomace (94.4%). Substantial differences were observed in biomass production (BM). Larvae reared on the standard diet showed the highest biomass production (100%), whereas those fed with goat manure and olive pomace exhibited significantly lower values, reaching 59.54% and 37.63%, respectively. In contrast, the mixture of olive pomace and goat manure (2:1) resulted in a biomass production of 98.68%, which was comparable to that obtained with the standard diet. The feed conversion rate (FCR) also differed among treatments. The lowest values were recorded for olive pomace (0.0109 ± 0.003) and goat manure (0.0181 ± 0.001), while the standard diet showed an FCR of 0.1228 ± 0.004. The olive pomace–goat manure mixture presented an intermediate FCR value (0.06481 ± 0.067), indicating an improved substrate conversion compared to the standard diet.

A similar trend was observed for substrate consumption (SC). The standard diet showed the highest consumption (58.86 ± 1.08%), followed by the olive pomace–goat manure mixture (39.79 ± 1.73%) and goat manure (30.39 ± 1.73%). Conversely, larvae fed with olive pomace alone exhibited very limited substrate consumption (3.03 ± 1.26%).

#### CHNS Content of the Frass from Bioconversion Assay

The elemental analysis showed differences in nitrogen (N), carbon (C), and hydrogen (H) content between the two frass samples obtained from different feeding substrates. The nitrogen content was higher in the Frass Standard Diet (2.98 ± 0.17%) compared to the Frass Olive pomace–goat manure 2:1 (2.16 ± 0.20%). Similarly, the carbon content was lower in the Frass Standard Diet (38.82 ± 0.30%) than in the Frass Olive pomace–goat manure 2:1 sample (42.82 ± 0.17%). Hydrogen content was higher in the Frass Standard Diet (5.42 ± 0.23%), whereas the Frass Olive pomace–goat manure 2:1 showed a lower value (4.66 ± 0.25%). Sulfur was not detected in either of the analysed samples (0.00%) (Table 3).

### 3.3. Anaerobic Digestion Assay

Two key parameters were evaluated in the anaerobic digestion process: the quantification of biogas production across the different treatments and the bioconversion efficiency of the larvae reared on the obtained digestate.

#### 3.3.1. Biogas Production

**Figure 1:**
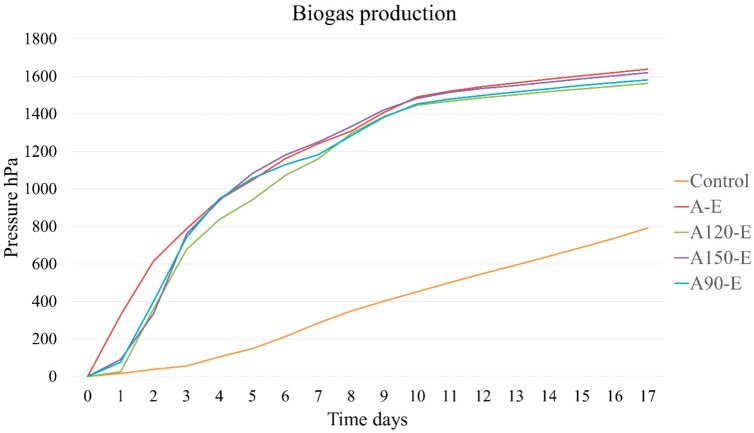
Cumulative biogas production in the different treatments during the anaerobic digestion period (37 °C). On the x-axis, the days of the digestion process are reported, while on the y-axis, the cumulative biogas pressure is expressed in hPa. Statistical differences were reported in Table 4.

The analysis of cumulative biogas production showed no significant differences among the different thermal pre-treatment conditions applied to olive pomace. A significant difference was observed only in comparison with the goat manure control. This control was specifically included to quantify the biogas production attributable exclusively to olive pomace, eliminating the potential contribution from the inoculum itself. The mixture of olive pomace + manure without pre-treatment achieved a biogas production similar to that of the three thermal pre-treatments. The line graph (Figure 1) shows the cumulative biogas production (expressed in hPa) during 17 days of anaerobic digestion, using five different substrates: A-E; A90-E, A120-E, A150-E and control. It can be seen that all pre-treatments had similar biogas production at the end of the experiment, indicating that thermal pre-treatment was not effective. The positive control produced approximately 500 hPa, ranking below all other treatments. The curves show a rapid increase in the first 10 days for all treatments, followed by a plateau indicating the depletion of the most easily degradable fraction.

It is noteworthy that the control treatment with manure alone showed a significantly lower performance compared to all other treatments. The greatest differences among treatments were observed during the first two days, where treatments A-E produced a significantly higher amount of biogas than the others. However, these differences among treatments were no longer significant after day three (Table 4).

##### CHNS Content of Digestates

Elemental analysis revealed significant differences in digestate composition depending on the thermal pretreatment applied. Nitrogen content was highest in A-E (2.38%) and lowest in A150-E (1.62%). Treatments at 90 °C and 120 °C showed values comparable to the control. Carbon content increased in all pretreated digestates compared to the control, reaching the highest value in A150-E (47.40%). Overall, moderate temperatures better preserved nitrogen content, whereas more severe treatments increased relative carbon content but reduced the fertilising potential of the digestate (Table 5).

#### 3.3.2. Bioconversion Assay on Digestates

The initial pH of the digestates used for larval feeding ranged between 7.0 and 7.5, indicating conditions suitable for larval development. Regarding the bioconversion process on digestates from the previous AD experiment, average larval weight, survival rate and FCR (Feed Conversion Rate), biomass and substrate consumption were measured.

The survival rate of *H. illucens* larvae (%) after nine days of growth on five different organic substrates (digestate) obtained from the anaerobic digestion process. The substrates tested included standard diet (SD) without anaerobic digestion, digested olive pomace with goat manure A-E, digested olive pomace pre-treated at 90 °C with goat manure (A90-E), digested olive pomace pre-treated at 120 °C with goat manure (A120-E), and digested olive pomace pre-treated at 150 °C with goat manure (A150-E) (Table 6). It is observed that commercial substrate (SD) had the highest survival rate, averaging 100% ± 0, followed by olive pomace at 120 °C (A120-E), which reached about 96.67% ± 2.31. Larvae reared on non-pretreated pomace (A-E) had a significantly lower survival rate of about 79.33% ± 7.57 (*p* < 0.05).

The average final fresh (wet) weights (expressed in grams) of *H. illucens* larvae fed for nine days with five different substrates: (SD), (A-E), (A90-E), (A120-E) and (A150-E) are reported in Table 6. Although all larvae were introduced with a similar initial fresh weight, at the end of the feeding period, differences in final fresh weight were observed depending on the substrate used. The larvae fed on the (SD) reached the highest weight, with an average final weight per larva of 0.16 g, followed by those fed (A-E), with an average weight per larva of 0.15 g. For the other three substrates (digestates), significantly less growth was obtained with an average weight per larva of 0.11 g (A90-E), 0.09 g (A120-E) and 0.11 g (A150-E), respectively.

The Feed conversion rate (g dw L/g dw S), calculated as the ratio of the larval biomass produced to the amount of substrate consumed, was tested in the five treatments. The larvae fed with the standard diet substrate (SD) showed the highest bioconversion efficiency (18.7%), followed by the non-pretreated olive pomace (A-E) with a value of about 15.3%. Olive pomace with the three different pre-treatments, on the other hand, showed a significantly lower bioconversion rate. The regression analysis for FCR yielded an R^2^ value of 0.5671, indicating that the model explains a moderate proportion of the observed variance (Table 6).

The highest biomass accumulation was observed in the treatment (SD), which generated 100% biomass, followed by (A-E) with 90%. Larvae fed with pre-treated pomace (A90-E), (A120-E) and (A150-E) produced 70%, 55% and 61% of the weight recorded in the SD, respectively. The total amount of larval biomass reflects the differences already observed in the individual growth and survival rate parameters (Table 6).

The SD treatment has the highest substrate consumption value, approximately 46–47%, clearly distinguishing itself from all other treatments. The substrates A-150E, A90-E and A120-E show no differences in substrate consumption, ranging from approximately 24% to 26%, suggesting a limited or comparable effect of heat pretreatment up to 120 °C on substrate consumption. The A150-E treatment shows slightly higher substrate consumption than the other pretreated substrates, with a value of approximately 29% (Table 6).

##### CHNS Content of the Frass from the Bioconversion Assay on Digestates

The elemental analysis revealed significant differences in nitrogen (N), carbon (C), and hydrogen (H) composition among the different frass samples derived from the digestates. The nitrogen content was highest in the Frass from Digestate control (2.72 ± 0.16%), followed by the Frass from Digestate A90-E (2.37 ± 0.15%). Intermediate values were observed in the Frass from Digestate A-E (1.94 ± 0.51%) and Frass from Digestate A150-E (1.54 ± 0.13%), whereas the lowest value was recorded in the Frass from Digestate A120-E (0.91 ± 0.14%). Regarding carbon, the highest content was found in the Frass from Digestate A-E (48.29 ± 0.32%), followed by samples A150-E (47.36 ± 0.34%) and A90-E (46.37 ± 0.41%). The control sample showed a lower value (38.27 ± 0.38%), while the lowest carbon content was observed in A120-E (35.91 ± 0.18%). Hydrogen content showed a trend similar to that of carbon, with the highest values in samples A150-E (5.08 ± 0.18%), A90-E (5.06 ± 0.17%), and A-E (5.02 ± 0.16%). The control exhibited an intermediate value (4.26 ± 0.26%), whereas sample A120-E presented the lowest content (3.26 ± 0.38%). Sulfur was not detected in any of the analysed samples (0.00%) (Table 7).

### 3.4. Phytotoxicity Tests

**Figure 2:**
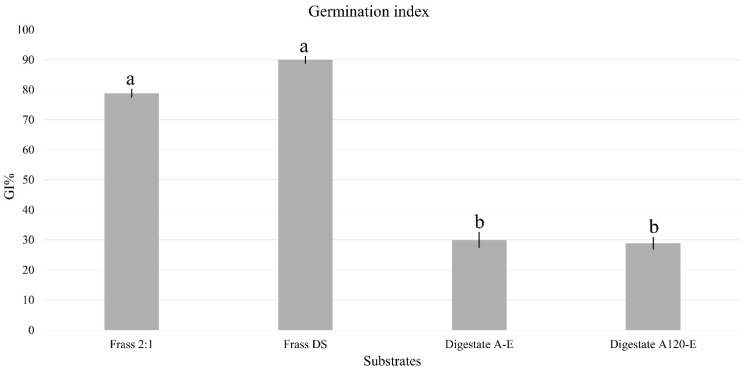
Phytotoxicity tests. The graph shows the Germination Index (GI) for the four substrates. The bars represent the mean ± standard deviation (*n* = 3 replicates). Different superscript letters (^a^, ^b^) denote significant differences within treatments (ANOVA test with *p* < 0.05).

The phytotoxicity of the substrates was first evaluated through the Germination Index (GI%), which accounts for both seed germination and root elongation. The results revealed a clear and significant difference between the two types of organic processing, regardless of the specific substrate composition (Figure 2). Both frass samples (Frass DS and Frass 2:1) reached the highest GI values (between 80% and 90%), with no significant difference between the standard diet and the olive pomace-based frass. Similarly, both digestates (Digestate A-E and Digestate A120-E) showed a sharp and comparable decline in GI% (approximately 30%).

**Figure 3:**
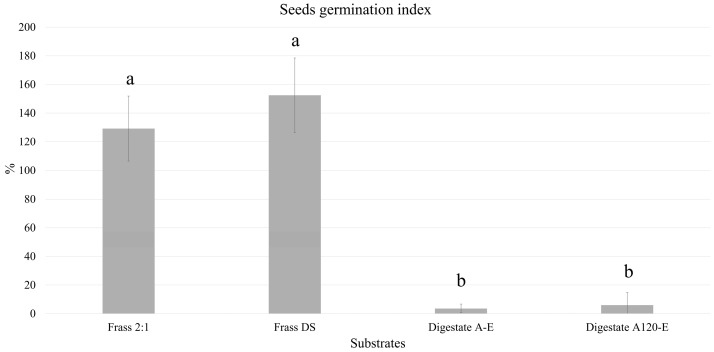
Phytotoxicity tests. The graph shows the Seeds Germination Index (SGI) for the four substrates. The bars represent the mean ± standard deviation (*n* = 3 replicates). Different superscript letters (^a^, ^b^) denote significant differences within treatments (ANOVA test with *p* < 0.05).

These findings are further supported by the Seeds Germination Index (SGI%) results (Figure 3). The frass from the control standard diet (Frass DS) showed the highest SGI (152.52% ± 25.97), indicative of total absence of phytotoxicity. The olive pomace/manure (2:1) frass (Frass 2:1) also showed SGI above 129.23% ± 22.69, suggesting good stability and maturity of the material. In contrast, the digestate of non-pretreated olive pomace (Digestate A-E) showed a moderately lower value (3.75% ± 3.13), while the digestate of pomace pre-treated at 120 °C (Digestate A120-E) showed a marked phytotoxic effect, with an SGI of 6.10% ± 8.73.

Digestates from untreated and treated pomace can inhibit germination and root growth. In contrast, processes such as the production of frass from the same raw material help reduce toxic compounds and improve the agronomic quality of the product.

## 4. Discussion

**Figure 4:**
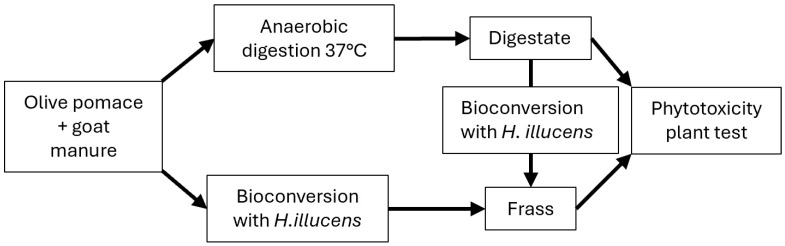
Schematic and summarising figure of the integrated approach used in the present study.

The integrated approach proposed in this study (Figure 4) provides valuable insights into the feasibility of converting agri-food wastes into value-added products, including renewable energy, sustainable fertilisers such as digestate and frass, and larval biomass [21,22,23,24,25]. This approach contributes to the advancement of circular economy strategies in the agri-food sector.

Larval performance is strongly influenced by the nutritional composition, physical structure, and microbial characteristics of the feeding substrate [26]. The relatively high R^2^ value observed for FCR suggests that a large proportion of the variability in conversion efficiency is explained by the tested substrate parameters, confirming the strong dependence of larval performance on substrate characteristics. Overall, the combination of olive pomace with goat manure leads to performance levels statistically comparable to those observed with the standard diet.

The elemental composition of frass reflects both the characteristics of the initial substrate and the metabolic activity of the larvae. In general, frass obtained from *H. illucens* contains around 2% nitrogen, depending on the feeding substrate [7,27], while variations in carbon and hydrogen content are closely linked to substrate composition and bioconversion dynamics [28]. Notably, there is a significant lack of data in the literature regarding the sulfur content of BSFL frass. To the best of our knowledge, one of the few studies addressing this, is by Romano et al., 2022 [29], who reported sulfur concentrations of 0.74% and 0.77% for fish-feed and cardboard-supplemented diets, respectively. In contrast, the sulfur content in the present study was found to be zero, further highlighting how drastically the mineral profile can vary or result in total depletion depending on the specific rearing substrate used. Another factor that may significantly influence the chemical composition of frass is the heat treatment process. According to European Regulation 1925/2021, frass must be treated at 70 °C for one hour to ensure biological safety and reduce the presence of pathogens such as *Salmonella* spp., *Escherichia coli*, and *Enterococcaceae* [30]. However, this thermal treatment can negatively affect the nitrogen profile, particularly the ammonia fraction. In a previous study [31], a comparison between treated and untreated frass revealed a reduction in ammoniacal nitrogen content following the 70 °C treatment. While this represents a limitation in terms of nutrient retention, it is a mandatory requirement to comply with legal standards for commercialisation and environmental safety. At the same time, although the treatment reduces ammonia content, it may also be beneficial in lowering potential phytotoxic effects on seed germination associated with excessive ammonia levels.

In addition to its nutrient content, frass may act as a carrier of beneficial microorganisms with plant growth–promoting properties, thereby enhancing soil biological activity [32].

The effect of thermal pretreatment of olive pomace (90, 120, and 150 °C) on anaerobic digestion performance was evaluated in terms of biogas production [32]. Contrary to expectations, thermal pretreatment did not significantly improve the process. Pretreatment technologies are commonly applied to lignocellulosic substrates to enhance hydrolysis and increase methane yields by disrupting plant biomass structure and promoting the solubilisation of organic compounds [18]; however, their effectiveness strongly depends on substrate properties and process conditions [31,33]. In the present study, untreated olive pomace mixed with inoculum showed comparable, and in some cases higher, cumulative biogas production than thermally pretreated substrates. Co-digestion of olive-mill waste with goat manure has been shown to enhance biogas production and organic matter removal. This suggests that mixing with manure can improve microbial accessibility and reduce the need for thermal pretreatment [34]. These findings indicate that olive pomace may already provide sufficient microbial accessibility under mesophilic conditions, thereby reducing the benefits of additional thermal processing. From a practical perspective, this suggests that energy-intensive pretreatment steps may not be necessary for the efficient anaerobic digestion of this substrate [35]. This aspect is also relevant from an economic standpoint, as avoiding energy-intensive pretreatments can significantly reduce operational costs without compromising overall system performance. Importantly, these observations have direct implications for downstream processing within the integrated system. Any modification in digestate composition resulting from pretreatment is likely to influence its subsequent use as a substrate for insect-based bioconversion.

Thermal pretreatment also influenced the chemical composition of the resulting digestates. A progressive reduction in nitrogen content was observed with increasing pretreatment temperature, reaching the lowest values in digestate derived from pomace treated at 150 °C.

This trend likely reflects nitrogen losses due to volatilisation or the thermal transformation of organic nitrogen compounds during heating, in agreement with previous studies on thermally treated organic residues [36,37]. Conversely, higher carbon contents were observed in digestates derived from thermally treated pomace, suggesting the accumulation of more recalcitrant carbon fractions formed during thermal processing. This may influence both agronomic value and suitability for subsequent biological processes. In particular, these compositional shifts are expected to play a key role in determining the efficiency of the subsequent larval rearing phase, linking anaerobic digestion performance with insect-based bioconversion outcomes.

Within the integrated anaerobic digestion–insect bioconversion system, the suitability of digestates as feed substrates represents a critical link between upstream processing and downstream biomass production. The results showed that digestate-based rearing conditions resulted in lower larval performance under the tested experimental setup, suggesting that performance could be further improved through optimisation of substrate properties and rearing conditions. Larvae reared on these substrates exhibited lower individual weight, reduced survival rates, and decreased substrate conversion efficiency compared with larvae fed digestate derived from untreated pomace. These findings suggest a cascade effect, whereby pretreatment-induced changes in substrate properties negatively propagate through the system, ultimately reducing the efficiency of larval bioconversion [38]. Moreover, these findings highlight the strong dependence of larval performance on substrate quality. Thermal treatment may alter nutrient availability, modify physical structure, or generate compounds that negatively affect larval feeding behaviour and metabolism [26].

It should be noted that larvae reared on digestates were maintained directly within the AD reactors, whereas larvae fed raw substrates were reared in trays. Therefore, the observed differences in performance may not be solely attributable to substrate characteristics but could also be influenced by differences in rearing conditions, such as aeration, physical structure, and microenvironment. This represents a limitation of the present study and should be addressed in future work through experiments conducted under comparable rearing conditions. Comparing these results with previous studies remains challenging due to different experimental setups. However, despite these variations, it is evident that BSF larvae can successfully develop on both substrates, confirming digestate as a viable option for larvae rearing.

However, this limitation should be interpreted in light of the broader BSF literature. Recent studies and reviews show that larval performance is strongly shaped by rearing conditions, including substrate type, substrate depth, aeration, moisture, larval density and overall protocol design, and that results are often difficult to compare across laboratories because study protocols differ [39,40,41]. This is directly relevant here, as recent work on digestate and other low-value residues shows that BSF outcomes depend not only on substrate composition, but also on whether the material is pretreated or processed under controlled conditions; for example, larvae fed hydrolyzed digestate grew better than those fed crude digestate, although still less efficiently than larvae on a standard diet, and larvae reared on biogas digestate achieved only a modest increase in weight [9,26]. Similar results have been reported for other organic residues, where the rearing system itself changed growth and conversion efficiency, and insufficient aeration on pasty substrates reduced performance [42]. Therefore, the differences observed in the present study cannot be attributed solely to the intrinsic suitability of digestate as a substrate, but may also reflect the rearing microenvironment, especially aeration, physical structure and handling conditions. This is why no definitive conclusion can be drawn about digestate under standard rearing conditions, and why standardised experimental designs are needed before cross-study comparisons can be treated as conclusive [39,41].

The elemental composition of frass produced from digestates further reflected the influence of the initial substrate. Frass derived from larvae fed digestate obtained from untreated pomace generally showed higher nitrogen concentrations compared with frass obtained from thermally pretreated substrates. This confirms that upstream processing steps can influence the chemical characteristics of downstream products, including frass. Variations in carbon and hydrogen content further support the link between substrate composition and bioconversion outcomes [43]. These findings further confirm that upstream processing decisions directly shape the quality of downstream products, reinforcing the interconnected nature of the system.

The choice of substrate is a primary driver of both larval performance and frass quality. Our results align with recent findings suggesting that *H. illucens* larvae can thrive on a wide range of alternative organic streams, though the specific substrate significantly dictates their nutritional profile [44]. In the context of integrated systems, substrate selection therefore represents a central factor influencing not only larval growth but also the overall efficiency and sustainability of the entire valorisation chain.

Phytotoxicity assays provided additional insights into the potential agricultural application of digestates and frass. Frass extracts exhibited high germination index values, indicating good maturity and the absence of significant phytotoxic effects [22,45]. In contrast, a clear and statistically significant difference was observed between frass and digestates, with the latter showing substantially lower germination indices, suggesting the presence of inhibitory compounds affecting seed germination and root development. Digestates, indeed, showed lower germination indices, suggesting the presence of compounds that may inhibit seed germination or root elongation [44,45]. These effects are commonly associated with elevated concentrations of ammonium, volatile fatty acids, or intermediate compounds formed during anaerobic digestion [46,47]. These findings indicate that the treatment and processing of the substrate significantly influence its residual phytotoxicity. The positive performance of frass is consistent with previous studies highlighting its potential as a sustainable organic fertiliser capable of improving plant growth and soil quality [30,48]. Recent reviews also emphasise the role of *H. illucens* frass as a valuable amendment within circular bioeconomy strategies [43,49].

Phytotoxicity assays provided additional insights into the potential agricultural use of digestates and frass. Recent digestate studies indicate a clear dose-dependent effect: digestates from food waste showed a germination index above 120% at a 5% dose, whereas higher concentrations were less suitable for plant establishment, and digestates from faecal sludge produced germination indices of 137% in tomato and 82% in cabbage [50,51]. A more recent full-scale study further showed that total ammonia nitrogen, potassium, and boron were the main predictors of phytotoxicity, with germination indices dropping below 50% when all thresholds were exceeded [52]. Frass showed similarly feedstock-dependent behaviour: frass from nine edible insects reached a germination index of 267%, but frass from the other species showed medium to high phytotoxicity [53,54]. In a separate study, frass derived from food waste had the lowest phytotoxicity, whereas frass from biosolids and wheat bran was more phytotoxic, and mealworm frass performed best at low doses of 0.5–1% in seedling production [55,56].

This highlights how the effects of upstream treatments extend beyond bioconversion efficiency to influence the final agronomic performance of the derived products.

Overall, the results highlight the importance of evaluating waste valorisation strategies from a system-level perspective. Process modifications applied at early stages, such as thermal pretreatment, can generate cascading effects that influence not only anaerobic digestion performance but also overall production costs, larval development and the quality of the resulting frass. These interdependencies underscore the need to optimise the entire process chain rather than individual steps in isolation.

Taken together, the findings of this study indicate that the direct use of untreated olive pomace represents the most efficient option within the investigated integrated system. From an economic perspective, these findings are particularly relevant. Although certain processing strategies, such as thermal pretreatment, are commonly applied to enhance process performance, they involve additional energy requirements and operational costs. In contrast, the direct use of untreated olive pomace reduces process complexity and avoids extra energy inputs. Therefore, even in scenarios where process yields are comparable or only marginally improved, the overall system may be more economically advantageous when low-cost, untreated substrates are used. This highlights the importance of considering not only technical efficiency but also economic sustainability when evaluating integrated waste valorisation systems.

This approach avoids additional energy inputs associated with thermal pretreatment while maintaining adequate performance in both anaerobic digestion and insect bioconversion. Thermal pretreatment did not provide clear benefits for anaerobic digestion and appeared to negatively affect the subsequent larval bioconversion process. These results underline the importance of carefully evaluating the interactions between pretreatment technologies, microbial processes, and insect-based bioconversion when designing integrated waste management strategies.

The combination of anaerobic digestion and bioconversion by *H. illucens* larvae represents a promising approach for transforming agro-industrial residues into renewable energy and value-added bio-products such as insect biomass and frass-based fertilisers (Figure 4). Such integrated systems have the potential to contribute significantly to sustainable resource management and circular bioeconomy models [5]. These results also suggest that economic optimisation may favour simpler process configurations, particularly when low-cost agro-industrial residues are available in large quantities.

## 5. Conclusions

This study investigated the integrated valorisation of olive pomace through anaerobic digestion and bioconversion by *Hermetia illucens*. Experimental results indicated that thermal pretreatment (90–150 °C) did not enhance biogas production and, in some cases, negatively affected nutrient composition and process performance. Under the tested conditions, the untreated substrate showed the best overall performance in terms of energy recovery. The resulting frass exhibited no phytotoxic effects and promising agronomic properties, whereas digestates showed lower germination indices, indicating the need for further stabilisation. Overall, the direct use of untreated olive pomace appears to be a more efficient configuration within the proposed system. Future research should focus on pilot-scale validation and comprehensive environmental and economic assessments to evaluate the feasibility and scalability of this approach under real industrial conditions.

## Figures and Tables

**Table 1:** Chemical–physical properties of the tested substrates (olive pomace, goat manure, and their 2:1 mixture), expressed on a dry matter basis.

Analysis	Olive Pomace	Goat Manure	Mixture (2:1)	R^2^
pH	6.10 ± 0.01 ^a^	8.87 ± 0.02 ^b^	7.48 ± 0.02 ^c^	0.9824
Electrical conductivity (mS/cm)	5.18 ± 0.05 ^a^	13.19 ± 0.29 ^b^	9.18 ± 0.06 ^c^	0.9713
Organic carbon %	5.71 ± 0.93 ^a^	3.62 ± 0.67 ^b^	4.66 ± 0.36 ^ab^	0.7264
Organic matter %	9.85 ± 1.60 ^a^	6.23 ± 1.16 ^b^	8.04 ± 0.62 ^ab^	0.6887
Volatile solids %	6.20 ± 0.62 ^a^	5.22 ± 0.21 ^b^	5.71 ± 0.24 ^ab^	0.7827
Nitrogen %	1.80 ± 0.25 ^a^	2.30 ± 0.15 ^b^	2.15 ± 0.10 ^ab^	0.7741
Phosphorus %	0.36 ± 0.12 ^a^	2.15 ± 0.21 ^b^	1.25 ± 0.22 ^c^	0.9463
Potassium %	2.14 ± 0.09 ^a^	1.71 ± 0.14 ^b^	1.92 ± 0.07 ^c^	0.9177

Values are expressed as mean ± standard deviation (*n* = 3). Data were subjected to one-way analysis of variance (ANOVA) followed by Tukey’s *post hoc* test for multiple comparisons. Different superscript letters (^a^, ^b^, ^c^) within the same column indicate statistically significant differences between groups (*p* < 0.05).

**Table 2:** Bioconversion parameters on larvae fed on a standard diet and a mix of olive pomace-goat manure 2:1. Survival rate (SR %), Feed Conversion Rate (FCR), Biomass production (BM %) and Substrate consumption (SC %).

Sample	SR (%)	FCR	BM (%)	SC (%)
Standard diet	98.4 ± 2.19 ^a^	0.1228 ± 0.004 ^a^	100 ^a^	58.855 ± 1.07 ^a^
Goat manure	97.6 ± 2.60 ^a^	0.0181 ± 0.001 ^b^	59.5420 ± 5.53 ^b^	30.3920 ± 1.72 ^c^
Olive pomace	94.4 ± 5.36 ^a^	0.0109 ± 0.003 ^b^	37.629 ± 3.89 ^c^	3.0320 ± 1.26 ^d^
Olive pomace-goat manure 2:1	98.3 ± 2.84 ^a^	0.0648 ± 0.067 ^ab^	98.6771 ± 2.89 ^a^	39.7898 ± 1.73 ^b^
R^2^	0.2483	0.7265	0.9873	0.9964

Values are expressed as mean ± standard deviation (*n* = 3). Data were subjected to one-way analysis of variance (ANOVA) followed by Tukey’s *post hoc* test for multiple comparisons. Different superscript letters (^a^, ^b^, ^c^, ^d^) within the same column indicate statistically significant differences between groups (*p* < 0.05).

**Table 3:** CHNS of frass from each raw material. Chemical properties of the tested frass are expressed on a dry matter basis.

Sample	Carbon %	Hydrogen %	Nitrogen %	Sulfur %
Frass Standard Diet	38.82 ± 0.29 ^b^	5.42 ± 0.23 ^a^	2.98 ± 0.17 ^a^	0.00
Frass mixture Olive pomace-goat manure 2:1	42.82 ± 0.17 ^a^	4.66 ± 0.25 ^b^	2.16 ± 0.19 ^b^	0.00

Values are expressed as mean ± standard deviation (*n* = 3). Data were subjected to one-way analysis of variance (ANOVA) followed by Tukey’s *post hoc* test for multiple comparisons. Different superscript letters (^a^, ^b^) within the same column indicate statistically significant differences between groups (*p* < 0.05).

**Table 4:** Statistical analysis of each individual day of the biogas production process.

Day	Goat Manure	A-E	A90-E	A120-E	A150-E	R^2^
0	0.00 ± 0	0.00 ± 0	0.00 ± 0	0.00 ± 0	0.00 ± 0	0.00
1	17.3 ± 1.56 ^b^	327.85 ± 71.8 ^a^	84.5 ± 15.3 ^b^	25.4 ± 8.75 ^b^	91.55 ± 1.75 ^b^	0.9472
2	38.6 ± 1.8 ^c^	615.35 ± 17.77 ^a^	392.85 ± 17.39 ^b^	361.4 ± 95.53 ^b^	337.35 ± 5.15 ^b^	0.9629
3	57.95 ± 2.85 ^b^	787.55 ± 17.21 ^a^	731.2 ± 19.61 ^a^	676.5 ± 100.7 ^a^	764.95 ± 11.05 ^a^	0.9811
4	107.4 ± 4.34 ^b^	943.5 ± 34.83 ^a^	939 ± 17.6 ^a^	837.3 ± 96 ^a^	944.55 ± 11.4 ^a^	0.9865
5	152.65 ± 5.87 ^d^	1047.85 ± 40.08 ^ab^	1049.8 ± 22.05 ^ac^	942 ± 92.3 ^bc^	1089.05 ± 11.86 ^a^	0.9887
6	216.6 ± 5.98 ^d^	1161.35 ± 47.35 ^ab^	1125.15 ± 18.24 ^ab^	1072.35 ± 79.03 ^bc^	1186.7 ± 11.85 ^a^	0.9913
7	288.85 ± 6.62 ^b^	1241.3 ± 51.77 ^a^	1189.1 ± 14 ^a^	1160.25 ± 84.66 ^a^	1255.75 ± 12.15 ^a^	0.9902
8	352 ± 6.2 ^b^	1308.6 ± 45.14 ^a^	1280.3 ± 18.04 ^a^	1296.05 ± 92.92 ^a^	1339 ± 12.9 ^a^	0.9899
9	405.25 ± 6.56 ^b^	1408.5 ± 30.80 ^a^	1376.1 ± 18.49 ^a^	1386.15 ± 83.47 ^a^	1429.10 ± 12.95 ^a^	0.9929
10	454.85 ± 6.36 ^b^	1490.4 ± 26.98 ^a^	1445.8 ± 15.87 ^a^	1447 ± 80.47 ^a^	1488.55 ± 12.10 ^a^	0.9938
11	504.2 ± 6.7 ^b^	1521.65 ± 23.37 ^a^	1472.10 ± 19.02 ^a^	1468.05 ± 75.22 ^a^	1521.65 ± 12.35 ^a^	0.9943
12	552.05 ± 6.75 ^b^	1545.85 ± 20.45 ^a^	1494.65 ± 16.5 ^a^	1486.6 ± 70.02 ^a^	1541.35 ± 11.6 ^a^	0.9949
13	597.1 ± 6.45 ^b^	1565.05 ± 18.97 ^a^	1513.95 ± 15.4 ^a^	1501.7 ± 69.14 ^a^	1557.5 ± 11.8 ^a^	0.9948
14	644.85 ± 7.2 ^b^	1585.45 ± 16.66 ^a^	1531 ± 15.18 ^a^	1519.1 ± 67.77 ^a^	1574.55 ± 11.75 ^a^	0.9947
15	692.15 ± 7.1 ^b^	1603.3 ± 14.15 ^a^	1549.55 ± 12.4 ^a^	1533.3 ± 67.2 ^a^	1592.95 ± 12.26 ^a^	0.9946
16	740.85 ± 6.85 ^b^	1620.2 ± 12.29 ^a^	1564.5 ± 11.91 ^a^	1547.3 ± 66.62 ^a^	1608.9 ± 12.16 ^a^	0.9943
17	794.9 ± 7.35 ^b^	1638.15 ± 11.46 ^a^	1585.8 ± 10.58 ^a^	1562.95 ± 65.54 ^a^	1625.4 ± 11.81 ^a^	0.9940

Values are expressed as mean ± standard deviation (*n* = 3). Data were subjected to one-way analysis of variance (ANOVA) followed by Tukey’s *post hoc* test for multiple comparisons. Different superscript letters (^a^, ^b^, ^c^, ^d^) within the same column indicate statistically significant differences between groups (*p* < 0.05).

**Table 5:** CHNS for each digestate. Chemical properties of the tested digestates are expressed on a dry matter basis.

Sample	Carbon %	Hydrogen %	Nitrogen %	Sulfur %
Digestate control	36.76 ± 0.50 ^a^	4.30 ± 0.25 ^b^	2.19 ± 0.01 ^a^	0.00
Digestate A-E	44.90 ± 0.61 ^c^	5.18 ± 0.14 ^a^	2.38 ± 0.04 ^b^	0.00
Digestate A120-E	42.46 ± 0.73 ^b^	4.96 ± 0.16 ^c^	2.18 ± 0.05 ^a^	0.00
Digestate A90-E	45.39 ± 0.69 ^c^	5.01 ± 0.08 ^c^	2.17 ± 0.03 ^a^	0.00
Digestate A150-E	47.40 ± 0.83 ^bd^	5.00 ± 0.13 ^c^	1.62 ± 0.09 ^c^	0.00
R^2^	0.9773	0.9845	0.9713	0.00

Values are expressed as mean ± standard deviation (*n* = 3). Data were subjected to one-way analysis of variance (ANOVA) followed by Tukey’s *post hoc* test for multiple comparisons. Different superscript letters (^a^, ^b^, ^c^, ^d^) within the same column indicate statistically significant differences between groups (*p* < 0.05).

**Table 6:** Bioconversion parameters on larvae fed on a standard diet and different digestates produced from a mixture of pre-treated and untreated olive pomace combined with goat manure. Survival rate (SR%), Feed Conversion Rate (FCR), Biomass production (BM%) and Substrate consumption (SC%).

Sample	SR (%)	Final Fresh Weight Per Larva	FCR	BM (%)	SC (%)
Standard diet	100 ± 0.00 ^a^	0.16 ± 0.001 ^a^	0.06 ± 0.005 ^a^	100 ± 0.00 ^a^	46.70 ± 5.48 ^a^
Digestate A-E	79.33 ± 7.57 ^b^	0.15 ± 0.003 ^ab^	0.06 ± 0.002 ^ab^	88.44 ± 2.68 ^ab^	24.27 ± 3.60 ^c^
Digestate A90-E	99.33 ± 1.15 ^a^	0.11 ± 0.02 ^bc^	0.05 ± 0.005 ^ab^	66.08 ± 18.41 ^bc^	25.84 ± 6.65 ^c^
Digestate A120-E	96.67 ± 2.30 ^a^	0.10 ± 0.004 ^c^	0.04 ± 0.002 ^b^	51.91 ± 4.07 ^c^	24.37 ± 1.34 ^c^
Digestate A150-E	92.33 ± 5.50 ^a^	0.11 ± 0.02 ^bc^	0.04 ± 0.02 ^b^	59.63 ± 10.71 ^c^	28.97 ± 8.39 ^bc^
R^2^	0.8213	0.8413	0.5671	0.8374	0.7730

Values are expressed as mean ± standard deviation (*n* = 3). Data were subjected to one-way analysis of variance (ANOVA) followed by Tukey’s *post hoc* test for multiple comparisons. Different superscript letters (^a^, ^b^, ^c^) within the same column indicate statistically significant differences between groups (*p* < 0.05).

**Table 7:** CHNS of frass from each digestate. Chemical properties of the tested frass were expressed on a dry matter basis.

Sample	Carbon %	Hydrogen %	Nitrogen %	Sulfur %
Frass from Digestate control	38.27 ± 0.38 ^c^	4.26 ± 0.26 ^b^	2.72 ± 0.16 ^a^	0.00
Frass from Digestate A-E	48.29 ± 0.32 ^a^	5.02 ± 0.16 ^a^	1.94 ± 0.51 ^b^	0.00
Frass from Digestate A120-E	35.91 ± 0.18 ^d^	3.26 ± 0.38 ^c^	0.91 ± 0.14 ^c^	0.00
Frass from Digestate A90-E	46.37 ± 0.41 ^b^	5.06 ± 0.17 ^a^	2.37 ± 0.15 ^ab^	0.00
Frass from Digestate A150-E	47.36 ± 0.34 ^ab^	5.08 ± 0.18 ^a^	1.54 ± 0.13 ^bc^	0.00
R^2^	0.9971	0.9264	0.8971	0.00

Values are expressed as mean ± standard deviation (*n* = 3). Data were subjected to one-way analysis of variance (ANOVA) followed by Tukey’s *post hoc* test for multiple comparisons. Different superscript letters (^a^, ^b^, ^c^, ^d^) within the same column indicate statistically significant differences between groups (*p* < 0.05).

## Data Availability

The original contributions presented in this study are included in the article. Further inquiries can be directed to the corresponding authors.
